# Suxiao Jiuxin Pill attenuates acute myocardial ischemia via regulation of coronary artery tone

**DOI:** 10.3389/fphar.2023.1104243

**Published:** 2023-05-10

**Authors:** Sa Li, Jiaguo Zhan, Yucheng Wang, Patrick Kwabena Oduro, Felix Boahen Owusu, Jiale Zhang, Ling Leng, Ruiqiao Li, Shujie Wei, Jun He, Qilong Wang

**Affiliations:** ^1^ Institute of Traditional Chinese Medicine, Tianjin University of Traditional Chinese Medicine, Tianjin, China; ^2^ State Key Laboratory of Component-Based Chinese Medicine, Ministry of Education, Tianjin, China; ^3^ Haihe Laboratory of Modern Chinese Medicine, Tianjin, China; ^4^ Endocrinology Department, Fourth Teaching Hospital of Tianjin University of Traditional Chinese Medicine, Tianjin, China

**Keywords:** Suxiao Jiuxin Pill, senkyunolide a, scopoletin, borneol, vasorelaxation, acute myocardial infarction

## Abstract

Suxiao Jiuxin Pill (SJP) is a well-known traditional Chinese medicine drug used to manage heart diseases. This study aimed at determining the pharmacological effects of SJP in acute myocardial infarction (AMI), and the molecular pathways its active compounds target to induce coronary artery vasorelaxation. Using the AMI rat model, SJP improved cardiac function and elevated ST segment. LC-MS and GC-MS detected twenty-eight non-volatile compounds and eleven volatile compounds in sera from SJP-treated rats. Network pharmacology analysis revealed eNOS and PTGS2 as the key drug targets. Indeed, SJP induced coronary artery relaxation via activation of the eNOS-NO pathway. Several of SJP’s main compounds, like senkyunolide A, scopoletin, and borneol, caused concentration-dependent coronary artery relaxation. Senkyunolide A and scopoletin increased eNOS and Akt phosphorylation in human umbilical vein endothelial cells (HUVECs). Molecular docking and surface plasmon resonance (SPR) revealed an interaction between senkynolide A/scopoletin and Akt. Vasodilation caused by senkyunolide A and scopoletin was inhibited by uprosertib (Akt inhibitor) and eNOS/sGC/PKG axis inhibitors. This suggests that senkyunolide A and scopoletin relax coronary arteries through the Akt-eNOS-NO pathway. In addition, borneol induced endothelium-independent vasorelaxation of the coronary artery. The K_v_ channel inhibitor 4-AP, K_Ca2+_ inhibitor TEA, and K_ir_ inhibitor BaCl_2_ significantly inhibited the vasorelaxant effect of borneol in the coronary artery. In conclusion, the results show that Suxiao Jiuxin Pill protects the heart against acute myocardial infarction.

## 1 Introduction

Acute myocardial ischemia (AMI) occurs due to an inadequate supply of oxygen to the myocardium due to narrowed coronary arteries. AMI causes angina, ischemic left ventricular dysfunction, arrhythmias, and myocardial necrosis. Vascular tone regulation is a complex multistep process (Jiang et al., 2015). The vascular endothelium is the innermost structure covering the lining of arteries, capillaries, and veins (Krüger-Genge et al., 2019). Endothelial cells contribute to the regulation of vascular tone by synthesizing and secreting prostacyclin (PGI_2_), nitric oxide (NO), and other endothelium-derived relaxation factors (Godo and Shimokawa, 2017). These relaxation factors maintain the balance of the intravascular environment and form the basis for treating coronary artery diseases. In clinical practice, vasodilators, such as nitrates and calcium channel blockers, are used to treat angina and acute coronary syndrome.

Suxiao Jiuxin Pill (SJP) is a well-known medicine in China used in the treatment of coronary heart diseases like angina pectoris, acute coronary syndrome, *etc*. SJP reduces the occurrence of angina attacks and helps alleviate stable and unstable angina symptoms (Duan et al., 2008). In patients with coronary heart disease, SJP improves electrocardiogram (ECG), lowers blood cholesterol, and regulates blood lipid profiles (Ren, L. et al., 2018). Pharmacological studies demonstrate that long-term administration of SJP protects against mitochondrial damage and alters damage-related gene expression in myocardial ischemic injury (Ruan et al., 2017), enhancing atherosclerotic plaque stability (Zhang, J. et al., 2014). Also, short-term administration of SJP could improve blood flow, reduce myocardial ischemia, and protects dogs from angina ischemia (Lu, Z. et al., 2015). In addition, SJP relaxes the human aorta in an endothelium-dependent manner (Bai et al., 2014).

SJP is composed of two components; *Ligusticum chuanxiong* Hort. and *Borneolum syntheticum*. The main chemical constituents are phthalides (e.g., senkyunolide A), phenolic acids (e.g., ferulic acid), alkaloids (e.g., ligustrazine), and borneol (Lei et al., 2018). Borneol exhibits cardioprotective effects by inhibiting apoptosis and reducing Ca^2+^ concentration (Liu, S. et al., 2021). Literature suggests that borneol relaxes the rat aorta in a dose-dependent manner, and K_ATP_ participates in its vasodilatory effect (Santos et al., 2019). Senkyunolide A, tetramethylpyrazine, ligustilide, and ferulic acid in *ligusticum chuanxiong* Hort. also show vasorelaxant effects (Shan Au et al., 2003; Cao et al., 2006; Chan et al., 2007; Zhou et al., 2017).

To understand the cardioprotective role of SJP, it is essential to have evidence of the pharmacological effects *in-vivo* and the molecular signaling pathways targeted by SJP and its active compounds. In this study, a biologically relevant AMI model and coronary artery vasorelaxation assays was used to elucidate the bioactive effects of SJP and identify the main bioactive compounds of SJP that stimulate the vasodilatory signaling pathways that protect against AMI.

## 2 Methods and materials

### 2.1 Chemicals and drugs

Suxiao Jiuxin Pill, *Ligusticum chuanxiong* Hort. [Apiaceae, Chuanxiong Rhizoma] extract and *Bornolum syntheticum* were provided by the Tianjin Darentang Group Co., Ltd., the sixth TCM factory (Tianjin, China). Voucher specimens were deposited in the Institute of Traditional Chinese Medicine, Tianjin University of Traditional Chinese Medicine. Scopoletin, senkyunolide H/I/A, sedanolide, ligustilide, angelicide, isochlorogenic acid C, vanillin, tetramethylpyrazine, isochlorogenic acid B, ferulic acid, caffeic acid, isochlorogenicacid A, cryptochlorogenic acid, protocatechuic acid, vanillic acid, neochlorogenic acid, succinic acid, chlorogenic acid, and isoborneol, (Z)-3-butylidenephthalide were purchased from Yuanye Bio-technology Co. Ltd. (Shanghai, China). Acetylcholine chloride (Ach), 9,11-dideoxy-11α, 9α-epoxy-methanoprostaglandin F_2α_ (U46619), N^G^-nitro-l-arginine methyl ester (L-NAME), indomethacin (INDO), methylene blue (MB), glibenclamide [Gli, ATP-sensitive K^+^ channels (K_ATP_) inhibitor], 4-aminopyridine [4-AP, voltage-dependent K^+^ channels (K_V_) inhibitor], tetraethylammonium [TEA, Ca^2+^-activated K^+^ channels (K_Ca2+_) inhibitor], BaCl_2_ [inward rectifier K+ channels (K_ir_)inhibitor], diltiazem (L-type calcium channel inhibitor), and dimethyl sulfoxide (DMSO) were purchased from Sigma-Aldrich (St. Louis, MO). HPLC grade acetonitrile was obtained from Fisher Scientific (Fair Lawn, NJ, USA). Potassium chloride (KCl) was bought from Guang Fu Technology Development Co. Ltd. (Tianjin, China). Uprosertib and 1H- [1,2,4] oxadiazolo [4,2-α] quinoxalin-1-one (ODQ) were acquired from MedChemExpress Co. Ltd. (Shanghai, China). All the chemicals were of analytical grade.

### 2.2 Animal treatment

Male Sprague-Dawley rats (200–250°g), 8–10°weeks old were purchased from SPF Biotechnology Company Ltd. (Beijing, China). All animals were kept in the Animal Center of Tianjin University of Traditional Chinese Medicine under standard conditions of 24°C–27°C, 60–70% relative humidity, and a 12 h light-dark cycle. All animals were fed a regular chow diet and water *ad libitum*. The Tianjin University of Traditional Chinese Medicine Institutional Animal Use and Care Committee approved all experimental procedures in this study with the following approval: No. TCM-LAEC2020079.

The rat AMI model was established by LAD ligation surgery (Li, J. et al., 2021). Rats were randomly divided into 5 groups: Sham, AMI model, SJP-IG group (54 mg/kg, intragastric gavage), SJP-IP group (54 mg/kg, intraperitoneal injection), and nitroglycerin group (0.0071 mg/kg, intragastric gavage). The SJP and nitroglycerin doses are the human equivalent doses calculated based on body surface area. SJP and nitroglycerin were administered immediately after ligation. Rats in both the sham and AMI groups received the same volume of saline.

### 2.3 Echocardiography

Transthoracic echocardiography was performed using an ultrasound system (Vinno, Suzhou, China) with a 22 MHz transducer at 5, 10, and 20 min after SJP and nitroglycerin treatment. The left ventricular internal dimension diastole (LVIDd), left ventricular internal dimension systole (LVIDs), left ventricular ejection fraction (EF), and fractional shortening (FS) were obtained with the heart rate controlled between 400 and 450 bpm. Three consecutive cardiac cycles were averaged to measure cardiac function.

### 2.4 Electrocardiogram (ECG)

SD rats were anesthetized and kept in the supine position. Needle electrodes were inserted subcutaneously according to lead II (right front leg, left hind leg, and left front leg) per the ECG protocol. ECG was recorded using PowerLab connected to BioAmp and analyzed by LabChart 8 software (AD instrument, Australia). The ECG signal was continuously monitored before ligation and for 20 min after drug administration. Changes in ECG patterns (ST segment) were analyzed.

### 2.5 Identification of chemical constituents in rat serum from SJP

#### 2.5.1 Sample preparation

Six SD rats were randomly divided into two groups after 7 days of acclimatization: the liquid chromatography-mass spectrometry (LC-MS) group (n = 3) and the gas chromatography-mass spectrometry (GC-MS) group (n = 3). Then, blood samples (300 µL) were collected (using heparinized centrifuge tubes) from the fossa orbitalis at pre-dose and 5 and 15 min after orally administered SJP at a 54 mg/kg dose. Blood samples were centrifuged immediately at 6000 g for 10 min at 4 °C, and the supernatants were transferred into a clean centrifuge tube. Each 100 μL sample was added to 500 μL of acetonitrile (LC-MS group) or 150 μL of n-hexane (GC-MS group) for protein precipitation and component extraction. The mixtures were centrifuged at 15,000 g for 10 min at 4 °C after being vortexed for 3 min. The GC-MS group obtained supernatants were stored at 4 °C before analysis. The LC-MS group obtained supernatants were transferred to clean 1.5 mL centrifuge tubes, followed by evaporation under a milt nitrogen stream. The obtained residues were individually redissolved in 100 µL of acetonitrile and centrifuged at 15,000 g for 10 min at 4 °C after being vortexed for 3 min. A volume of 10 µL of individual supernatants were then injected for analysis.

#### 2.5.2 LC-MS conditions for identification of non-volatile compounds in serum

Agilent 1290 and Q-TOF 6520 equipped with an X-select HSS T3 column (2.1 × 100 mm, 3.5 µm) were used to separate and detect non-volatile serum compounds after oral administration of SJP. The solvent system consisted of 0.1% formic acid aqueous solution A) and acetonitrile B). Electrospray Ionization Mass Spectrometry (ESI-MS) was set to both positive and negative ionization modes. The mass parameters were set as follows: capillary temperature, 350 °C; auxiliary gas rate, 11 L/h; spray voltage, 135 V; normalized collision energy, 40 V; and mass range, 150–1500 m*/z*.

#### 2.5.3 GC-MS analysis for identification of volatile compounds in serum

Detection of the volatile compounds of SJP in serum was performed on a Shimadzu GC-MS 2010 solution (Shimadzu, Japan) equipped with a DB-17 column (30 m × 0.25 mm × 0.25 μm). Helium was used as the carrier gas at a 1.4 mL/min flow rate. The ion source and interface temperatures were set at 230 °C and 250 °C respectively.

### 2.6 Network pharmacology analysis

Gene targets of 14 identified serum compounds were summarized from TCMSP (https://old.tcmspe.com/tcmsp.php), BATMAN-TCM (http://bionet.ncpsb.org.cn/batman-tcm/index.php), and Swiss Target Prediction (http://www.swisstargetprediction.ch/). AMI-associated targets were retrieved from OMIM (https://omim.org/), GeneCards (https://www.genecards.org/), and CTD (http://ctdbase.org/). Cytoscape 3.7.2 and DAVID (https://david.ncifcrf.gov/) were used to filter key targets and search for key compounds and pathways. The compounds-targets-pathway network was visualized through Sankey Tools (https://www.zxgj.cn/g/sankey).

### 2.7 Coronary arterial ring preparation and vascular reactivity assessment

The fresh heart was removed from SD rats and immediately immersed in a petri dish containing a K-H solution composed of NaCl 118 mmol/L, KCl 4.7 mmol/L, NaHCO_3_ 25 mmol/L, KH_2_PO_4_ 1.2 mmol/L, MgSO_4_ 1.2 mmol/L, CaCl_2_ 1.3 mmol/L, and d-glucose 10 mmol/L. The rat coronary arteries were carefully separated and cut into 2 mm rings. The coronary artery from one rat was cut into 2 segments for each sample measurement. The arterial ring was suspended in an organ bath of wire myograph (DMT A/S, Model 630MA, Denmark) containing K-H solution with an aerating mixture of 95% O_2_ and 5% CO_2_ at 37 °C.

After equilibration, arterial rings were constricted with U46619 (100 nmol/L) to the maximal contraction, and endothelial integrity was evaluated using cumulative concentrations of Ach (10-9–10–5 mol/L). The endothelium was considered intact when the relaxation rate was more than 80%. Endothelium-denuded rings were obtained by rubbing the inner surface of the artery several times with rat whiskers. Finally, the cumulative concentration-response curve for the SJP and its components was investigated. The EC_50_ and R_max_ (maximal vasorelaxation) values were then calculated.

### 2.8 Cell culture and western blotting analysis

Human umbilical vein endothelial cells (HUVECs) were purchased from AllCells Biotech Co., Ltd. (Shanghai, China). The cells were cultured in an Endothelial Cell Complete Culture Medium (Procell, Wuhan, China) containing 10% FBS and 1% penicillin/streptomycin at 37 °C in a humidified atmosphere of 5% CO_2_ and 95% air. The cells within 3-8 passages were used in the following experiments. The cells were serum-starved for 6 h, then treated with senkyunolide A or scopoletin for 15 min. Western blot analysis was performed using anti-eNOS (1:1000), anti-p-eNOS (1:1000, Ser1177), anti-Akt (1:1000), anti-p-Akt (1:1000, Thr308), and anti-GAPDH antibodies (1:1000) (CST, MA, USA).

### 2.9 Molecular docking

The X-ray 3D crystal structure of the human Akt1 pleckstrin homology (PH) domain, PDB ID: 1H10 (Thomas et al., 2002), was downloaded from the protein data bank repository (http://www.rcsb.org/). The 3D chemical structure of senkyunolide A, or scopoletin, was obtained from the PubChem database. The X-ray crystal protein preparation and the molecular docking were done using Discovery Studio Client (2019) and Autodock Vina, as described in our previous study (Fang et al., 2022), and the results were visualized with PyMOL.

### 2.10 Surface plasmon resonance (SPR) analysis

To evaluate the binding affinity of senkyunolide A or scopoletin to Akt, an SPR assay was performed using the Biacore T200 (Cytiva Corp., MA, USA). The recombinant human Akt1 protein (Bio-techne, Shanghai, China) was immobilized on an S CM5 sensor chip using standard amine-coupling chemistry. A serial dilution of the senkyunolide A (62.5, 125, 250, 500, and 1000 μmol/L) or scopoletin (5, 10, 20, 40, and 80 μmol/L) in running buffer was injected over the human Akt1 protein (1775-KS-010, Bio-Techne). With Biacore T200 evaluation software (Cytiva), the data fit into a 1:1 steady binding model to calculate the binding affinity KD.

### 2.11 Statistical analysis

SPSS version 21.0 for Windows (SPSS Inc., Chicago, IL, USA) was used for statistical analysis. All values are given as means ± standard error of the mean (S.E.M). Statistical significance was analyzed using one-way or two-way analysis of variance, followed by the Bonferroni *post hoc* analysis, and *p* < 0.05 was considered to be statistically significant.

## 3 Results

### 3.1 SJP improves cardiac function and ECG in AMI rats

**FIGURE 1:**
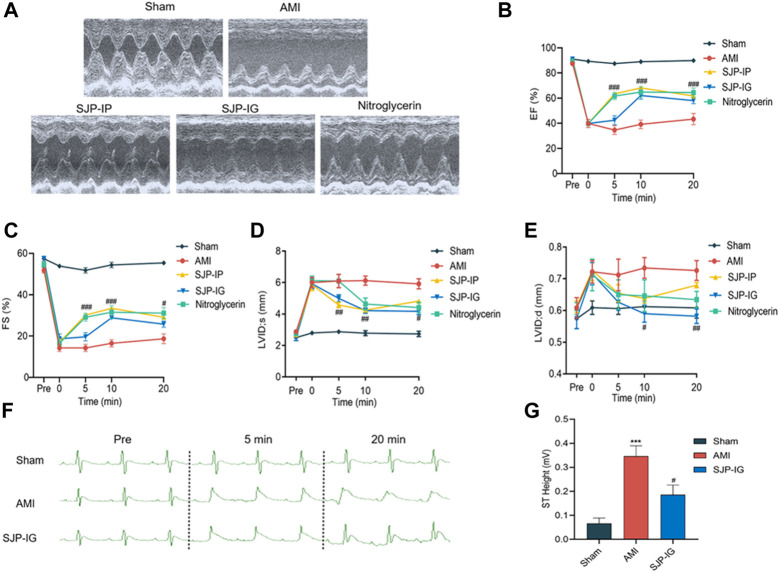
Evaluation of cardiac function and ECG in AMI rats treated with SJP. The rat AMI model was established by LAD ligation surgery. AMI rats were treated with SJP (54 mg/kg) by intragastric gavage (SJP-IG), intraperitoneal injection (SJP-IP), or nitroglycerin (0.0071 mg/kg) after LAD ligation. **(A)** Representative ultrasonic image of sham, AMI, SJP-IP, SJP-IG, and nitroglycerin group. AMI rat was administered with the drug for 20 min **(B–E)** Echocardiographic evaluation of cardiac function. Ejection fraction (EF), fractional shortening (FS), the left ventricular internal end-systole diameter (LVID, s), and the left ventricular internal end-diastolic diameter (LVID,d) were measured at 0, 5, 10, and 20 min of drugs administration. **(F)** Representative ECG images of sham, AMI, and SJP-IG group. **(G)** ST segment height in sham, AMI, and SJP-IG groups. N = 5; ***p < 0.001 vs. sham group; ^#^ *p* < 0.05, ^##^ *p* < 0.01, ^###^ *p* < 0.001 vs. AMI group.

To assess the therapeutic effect of SJP on angina, AMI was induced in rats by LAD ligation, after which cardiac function was examined. In the rat AMI model, EF and FS were significantly reduced, with an increase in LVIDs and LVIDd values after LAD ligation. SJP treatment (54 mg/kg) significantly reduced EF, FS, and LVID values 5–20 min after intraperitoneal injection and 10–20 min after intragastric gavage administration. Nitroglycerin, as a positive control, exhibited a similar effect on cardiac function (Figure 1A–E). The ST segment elevation in ECG was observed in the AMI model group, compared with sham control rats. However, SJP treatment significantly reduced the ST segment elevation caused by LAD ligation (Figures 1F,G).

### 3.2 Identification of chemical components in serum from SJP-treated rat

**FIGURE 2:**
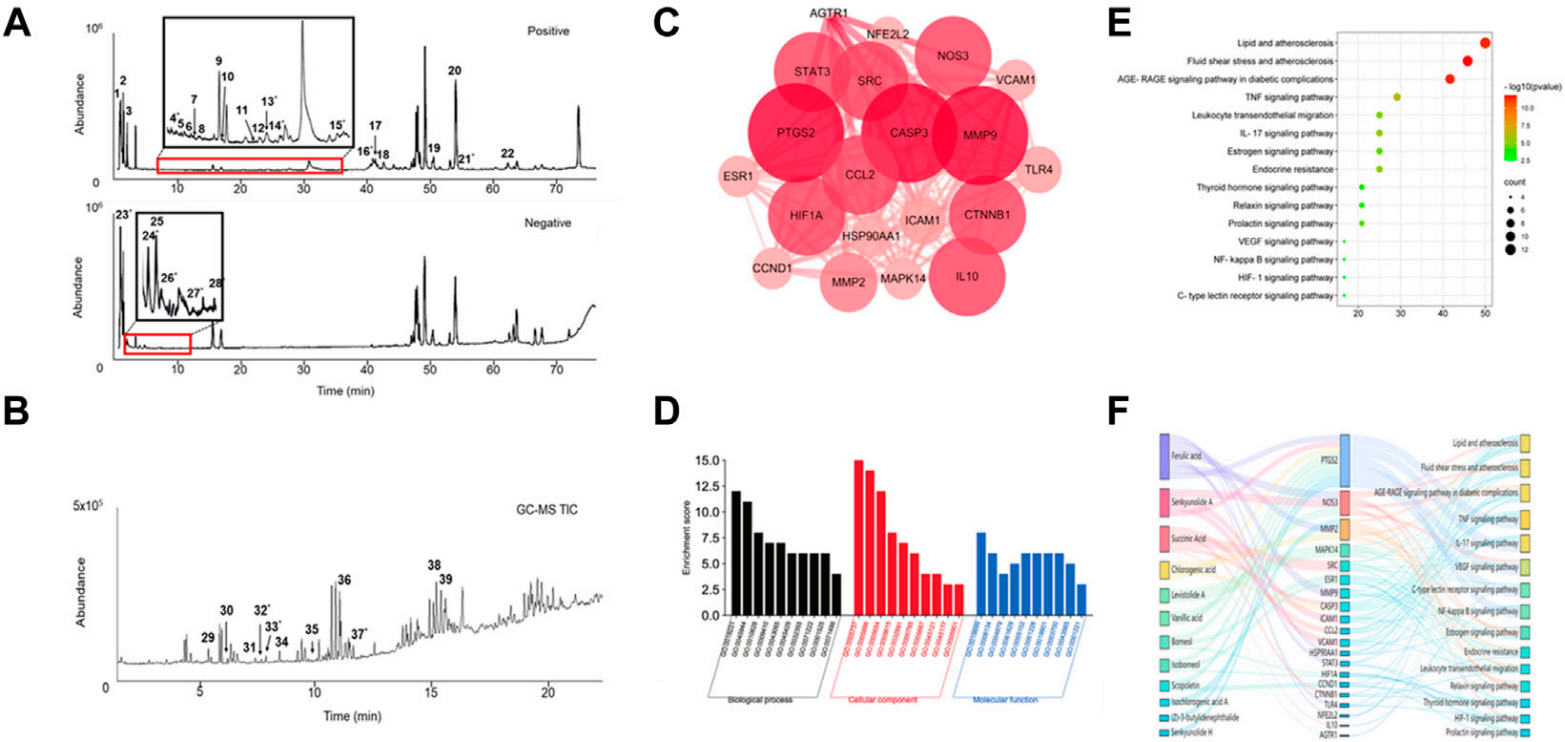
Chemical compounds found in serum and network pharmacology analysis. **(A)** The total ion current (TIC) chromatogram in both positive and negative modes for serum from SJP-treated rats. **(B)** The TIC chromatogram of GC-MS for serum from SJP-treated rats. **(C)** The PPI network of 20 intersection gene targets. **(D)** GO analysis. Enriched biological processes, cellular components, and molecular functions. **(E)** KEGG pathway analysis. A bubble diagram of differentially expressed genes. **(F)** The components-target-pathway interaction network of SJP improved AMI.

To identify the active ingredients of SJP, LC-MS, and GC-MS analysis were performed on serum from SJP-treated rats and Agilent Masshunter Qualitative Analysis was used to determine the identities of the various circulating compounds. The base peak chromatogram of the non-volatile compounds for the positive and negative ion modes is shown in Figure 2A. By comparing their retention time, mass-to-charge ratio, and secondary mass spectrum to their respective standard chemical compounds and previous phytochemical investigation reports, a total of 28 compounds were identified (Table 1). In addition, a total of 11 volatile circulating compounds were detected when standard chemical compounds were compared to the NIST database. The GC-MS TIC traces chromatogram is shown in Figure 2B and Table 2 lists the GC-MS profiling of the volatile circulating compounds.

**TABLE 1:** Identification of non-volatile metabolites in rat plasma after Suxiao Jiuxin Pill treatment by LC-MS in positive and negative ion mode.

No.	RT time	m/z	Ion mode	Formula	Compound name	ppm	ms/ms
1	0.94	144.1009	[M + H]^+^	C_7_H_13_O_2_N	Stachydrine	-7.41	84.0803
2	0.94	116.0694	[M + H]^+^	C_5_H_9_NO_2_	L-Proline	-9.56	70.0655
3	1.27	132.1007	[M + H]^+^	C_6_H_13_NO_2_	L-Isoleucine	-9.43	86.0964
4^a^	7.62	193.0497	[M + H]^+^	C_10_H_8_O_4_	Scopoletin	1.24	178.0345, 137.0690
5	8.98	227.1260	[M + H]^+^	C_12_H_18_O_4_	Senkyunolide J	-8.80	153.0535
6	10.32	207.1011	[M + H]^+^	C_12_H_14_O_3_	Butylidene phthalide	-8.79	161.0551, 189.0879
7	11.05	229.0814	[M + Na]^+^	C_12_H_14_O_3_	Senkyunolide F	-8.79	161.1055
8	13.26	227.1267	[M + H]^+^	C_12_H_18_O_4_	Senkyunolide N	-6.66	209.1125
9	15.86	163.0392	[M + H]^+^	C_9_H_6_O_3_	7-Hydroxycoumarine	1.37	107.0435
10	15.93	205.0854	[M + H]^+^	C_12_H_12_O_3_	7-Hydroxy-3-butylphthalide	-6.80	187.0755
11	22.51	229.0814	[M + Na]^+^	C_12_H_14_O_3_	(Z)-3-Butylidene-4-hydroxyphthalide	-8.79	189.0884, 161.0965
12	22.78	209.1157	[M + H]^+^	C_12_H_16_O_3_	Senkyunolide K	-5.62	191.1041, 153.0527
13^a^	24.12	247.0938	[M + Na]^+^	C_12_H_16_O_4_	Senkyunolide I	-2.85	207.1077
14^a^	25.25	247.0936	[M + Na]^+^	C_12_H_16_O_4_	Senkyunolide H	-3.06	207.1135
15^a^	36.91	193.1197	[M + H]^+^	C_12_H_16_O_2_	Senkyunolide A	1.03	175.1031, 147.1072, 137.0527
16^a^	40.25	191.1058	[M + H]^+^	C_12_H_14_O_2_	3-Butylphthalide	-8.95	173.0779, 145.1045
17	43.61	195.1363	[M + H]^+^	C_12_H_18_O_2_	Neocnidilide	-7.17	167.0143, 157.0126, 140.9554
18	43.70	191.1075	[M + H]^+^	C_12_H_14_O_2_	(Z)-Ligustilide	2.61	149.1282, 145.1045
19	52.04	383.2226	[M + H]^+^	C_24_H_30_O_4_	Senkyunolide P	1.93	191.1080
20	53.86	279.1579	[M + H]^+^	C_16_H_22_O_4_	Senkyunolide Q	-4.92	191.1017
21^a^	55.53	381.2045	[M + H]^+^	C_24_H_28_O_4_	Levistolide A	-4.71	335.2108, 191.1020
22	63.24	281.2463	[M + H]^+^	C_18_H_32_O_2_	Linoleic acid	-4.56	245.2246, 97.1001
23^a^	1.39	117.0191	[M-H]^-^	C_4_H_6_O_4_	Succinic acid	-1.14	72.9920
24^a^	4.22	353.0982	[M-H]^-^	C_16_H_18_O_9_	Chlorogenic acid	-7.92	136.9158, 130.9671, 96.9604
25	4.49	197.0475	[M-H]^-^	C_9_H_10_O_5_	Danshensu	4.68	135.0520
26^a^	5.02	167.0398	[M-H]^-^	C_8_H_8_O_4_	Vanillic acid	-4.19	112.9856, 104.9537
27^a^	8.27	193.0557	[M-H]^-^	C_10_H_10_O_4_	Ferulic acid	6.73	178.0365, 134.0368
28^a^	10.28	515.1181	[M-H]^-^	C_25_H_24_O_12_	Isochlorogenic acid A	4.85	353.0878, 191.0513

^a^ Compounds identified by comparison with reference standards.

**TABLE 2:** GC-MS TIC parameters on the identified volatile metabolites in plasma from rats treated with Suxiao Jiuxin Pill.

No.	RT (min)	Formula	Molecular weight (m/z)	Identification	SI	Retention index	Ratio (%)
29	6.785	C_10_H_22_	142.282	3-Ethyl-3-methyl heptane	92	931	0.48
30	7.588	C_13_H_28_	184.361	3,8-Dimethylundecane	94	1185	0.80
31	8.473	C_10_H_16_O	152.233	(±)-Camphor	93	1121	0.23
32^a^	8.690	C_10_H_18_O	154.249	Isoborneol	93	1138	0.17
33^a^	8.855	C_10_H_18_O	154.249	Borneol	94	1088	0.31
34	9.344	C_12_H_26_	170.335	Dodecane	96	1214	0.50
35	10.764	C_18_H_38_	254.494	Octadecane	93	1810	0.71
36	11.364	C_12_H_26_O	186.334	2-Butyl-1-octanol	88	1393	2.85
37^a^	12.170	C_12_H_12_O_2_	188.223	(Z)-3-Butylidenephthalide	87	1655	0.22
38	14.931	C_22_H_46_	310.601	Docosane	81	2208	0.39
39	15.321	C_13_H_28_O	200.361	Isotridecanol	86	1492	2.63

^a^ Compounds identified by comparison with reference standards.

### 3.3 Network pharmacology analysis for SJP targets on AMI

Network pharmacology was used to identify potential pharmacological targets of SJP in the treatment of AMI. A total of 14 compounds found in the serum of SJP-treated rats were mapped to databases of cellular drug targets, and a total of 20 genes, and 15 pathways were identified as a result. To better understand the differential relationship between SJP and AMI, a PPI network was constructed, which identified PTGS2, CASP3, MMP9, and NOS3 genes as the leading targets (Figure 2C). In addition to understanding the cellular connection between SJP and AMI, a pathway enrichment analysis was conducted on the key targets. The cellular pathways through which SJP may exert its beneficial therapeutic effect were identified as lipid and atherosclerosis, fluid shear stress, atherosclerosis, and AGE-RAGE signaling (Figures 2D,E). It was also uncovered that ferulic acid and senkyunolide A were all closely associated with the top two cellular drug targets, PTGS2 and NOS3 (Figure 2F). These findings, combined with the previous observations indicate that PTGS2 and NOS3 play a direct role in the regulation of endothelial function and vascular tone, reveal that SJP improves AMI by regulating artery relaxation.

### 3.4 Evaluation of the vasorelaxant effect of SJP on coronary artery

**FIGURE 3:**
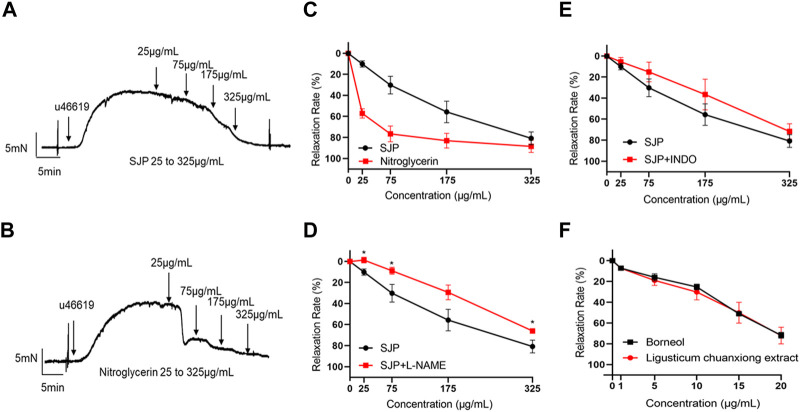
The vasorelaxant effect of SJP on coronary artery rings. **(A–B)** The representative tracing of a rat coronary artery treated with SJP **(A)** and nitroglycerin **(B)**. **(C)** The vasodilatory effects of SJP and nitroglycerin on rat coronary artery. **(D–E)** Relaxation curve of SJP on coronary arteries pre-contracted with U46619 in the presence of L-NAME (100 μmol/L) or INDO (10 μmol/L). **(F)** Vasorelaxation effects of *ligusticum chuanxiong* extract and borneol on U46619-induced vascular constriction in endothelium-intact rat coronary artery rings. N = 6, *p < 0.05 vs. no L-NAME treatment.

A test was conducted to ascertain whether SJP could dilate coronary artery rings. Results revealed that SJP has a concentration-dependent vasodilatory effect on the endothelium-intact coronary artery rings (EC_50_ = 136.1 μg/mL; R_max_ = 80.72 ± 10.27%). Nitroglycerin, as a positive control, relaxed the coronary artery with an EC_50_ of 22.1 μg/mL (Figures 3A–C).

In line with the network pharmacology findings, investigations into whether the NOS3 inhibitor L-NAME or PTGS2 inhibitor INDO could prevent SJP-induced coronary artery relaxation revealed that the relaxation effect of SJP was significantly suppressed by L-NAME treatment (R_max_ dropped from 80.72% to 65.96%) but not by INDO (Figures 3D,E), indicating that SJP modulates the eNOS-NO pathway to induce vasorelaxation.

### 3.5 Evaluation of vasorelaxant components of SJP

Extracts of the two active ingredients in SJP (*Ligusticum chuanxiong* Hort. and *Borneolum syntheticum*) had potent vasodilatory effects on the endothelium-intact coronary artery rings (Figure 3F). The findings inform that the active compound(s) of SJP is responsible for SJP’s vasorelaxation action. It was therefore tested to determine whether the compounds identified in the serum of SJP-treated mice, as well as other chemical compounds isolated from SJP (Lei et al., 2018; Li, S. L. et al., 2003; Liang et al., 2005), could respond to constricted coronary arteries in the vasoreactivity assay. All the compounds tested exhibited concentration-dependent vasodilation effects on coronary artery rings (Table 3). Scopoletin, sedanolide, butylphthalide, senkyunolide A, and borneol particularly had potent relaxation effects.

**TABLE 3:** Relaxation responses induced by the compounds of Suxiao Jiuxin Pill.

Chemical compound	Relaxation (%)					EC_50_ (μmol/L)
	0.1	1	10	100	1000 (μmol/L)	
Scopoletin	9.88 ± 0.89	48.26 ± 6.84**	75.00 ± 9.69**	91.03 ± 4.68**	96.58 ± 2.76**	1.51
Sedanolide	13.68 ± 1.69*	23.24 ± 3.63**	32.45 ± 6.88*	60.87 ± 11.51**	81.73 ± 17.26**	33.67
Butylphthalide	6.37 ± 6.11	11.2 ± 6.30	28.24 ± 14.28	62.59 ± 9.48**	97.51 ± 6.27**	37.42
Senkyunolide A	6.12 ± 1.4	14.84 ± 6.19	27.31 ± 6.05*	60.37 ± 13.94*	101.78 ± 8.03**	37.82
Ligustilide	5.02 ± 2.99	14.32 ± 3.15	23.07 ± 6.78	50.75 ± 8.01**	89.66 ± 1.82**	67.97
Borneol	7.45 ± 0.65	20.52 ± 7.37	27.39 ± 9.55	48.19 ± 17.46*	83.63 ± 7.00**	119.7
Senkyunolide H	5.28 ± 0.01	17.27 ± 13.25	25.44 ± 13.48	34.65 ± 11.60	73.82 ± 9.47**	174.1
Angelicide	4.99 ± 2.11	16.23 ± 1.22**	27.16 ± 5.35*	42.76 ± 13.45	63.34 ± 12.09**	207.1
Isochlorogenic acid C	3.35 ± 3.69	8.74 ± 1.75	13.23 ± 3.05	21.69 ± 8.21	73.41 ± 14.53**	339.8
Vanillin	3.06 ± 2.00	6.24 ± 4.43	14.83 ± 2.90	22.36 ± 3.28	64.60 ± 5.85**	455.3
Tetramethylpyrazine	3.84 ± 2.77	7.18 ± 2.65	11.33 ± 3.37	19.68 ± 6.00	60.82 ± 10.88**	576.9
Isochlorogenic acid B	3.36 ± 1.54	11.33 ± 9.36	19.37 ± 17.40	22.45 ± 18.93	59.64 ± 15.43*	625.6
Ferulic acid	5.20 ± 2.87	10.93 ± 4.6	17.55 ± 8.13	22.39 ± 6.07	47.72 ± 13.62	>1000
Caffeic acid	6.94 ± 2.83	11.25 ± 7.15	14.30 ± 8.15	21.24 ± 9.73	46.30 ± 9.39*	>1000
Isochlorogenicacid A	6.84 ± 2.73	9.88 ± 1.75	12.04 ± 2.24	25.78 ± 12.43	39.31 ± 14.26	>1000
Cryptochlorogenic acid	9.00 ± 4.21	16.22 ± 5.81	22.30 ± 7.14	28.26 ± 10.89	36.69 ± 15.30	>1000
Protocatechuic acid	6.67 ± 3.83	10.97 ± 6.07	15.38 ± 10.74	23.35 ± 9.90	32.53 ± 6.60	>1000
Vanillic acid	2.88 ± 7.10	9.12 ± 9.84	11.57 ± 15.02	17.75 ± 16.48	29.79 ± 13.03	>1000
Neochlorogenic acid	0.76 ± 1.80	4.24 ± 0.25	10.08 ± 3.89	15.74 ± 7.73	25.86 ± 9.46	>1000
Senkyunolide I	1.79 ± 4.37	5.56 ± 4.10	9.39 ± 5.40	18.22 ± 5.60	23.27 ± 7.67	>1000
Succinic acid	3.41 ± 2.65	6.19 ± 2.92	11.35 ± 4.01	13.33 ± 8.39	22.50 ± 10.24	>1000
Chlorogenic acid	7.38 ± 2.12	14.97 ± 6.15	20.57 ± 4.39	24.96 ± 5.53	22.27 ± 6.72	>1000
Acetylcholine	3.98 ± 4.74	14.82 ± 13.29	97.02 ± 5.54**			2.16

N = 6, *p < 0.05, **p < 0.01 vs. DMSO.

### 3.6 Vasorelaxation action mechanisms of senkyunolide a and scopoletin

**FIGURE 4:**
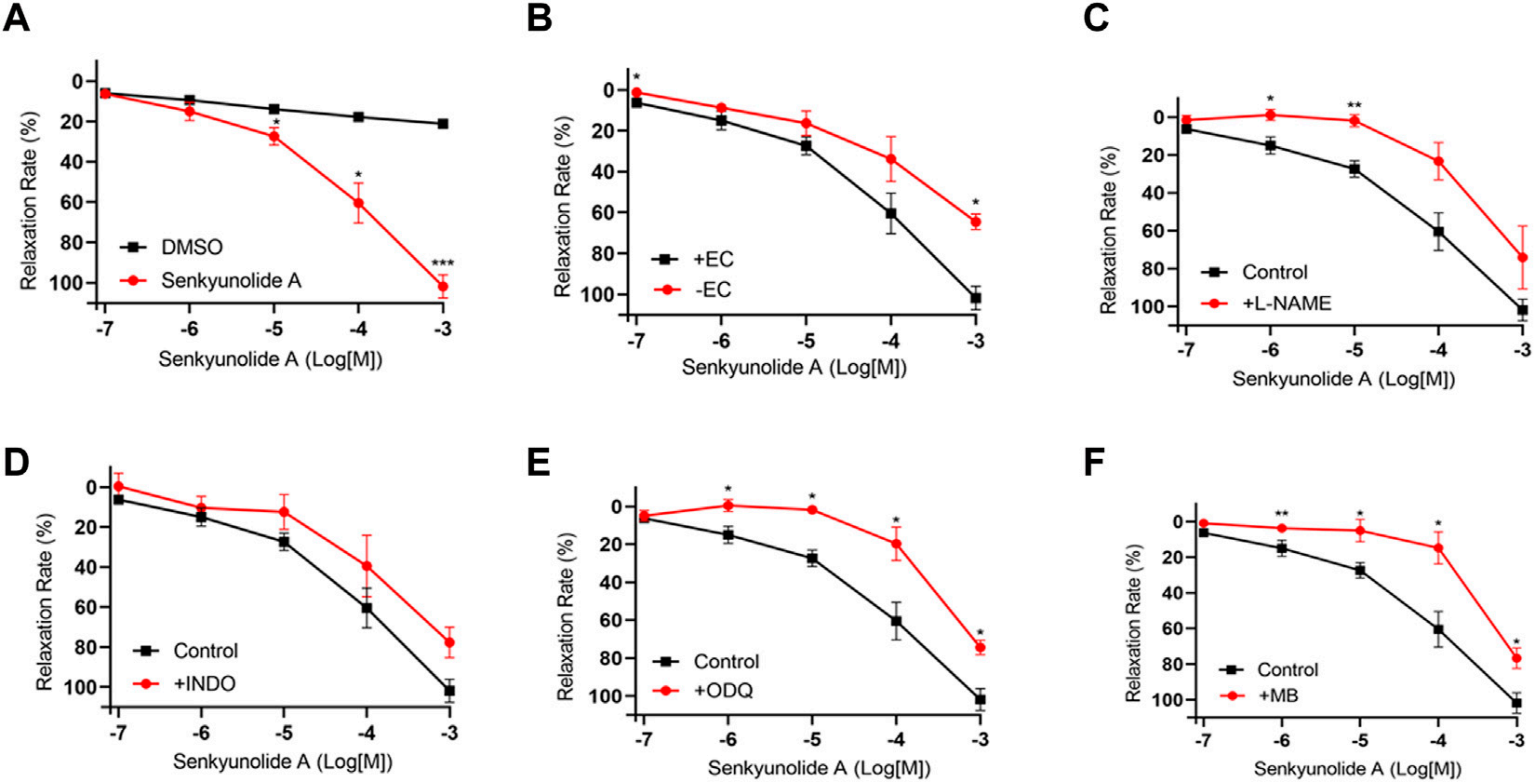
Endothelium-dependent mechanism of the vasodilation action of senkyunolide **(A) (A)** Relaxation curve of senkyunolide **(A) (B)** Relaxation curve of senkyunolide A on endothelium-intact (+EC) and endothelium-denuded (-EC) coronary artery rings. **(C–F)** Relaxation curve of senkyunolide A on coronary artery ring pretreatment with eNOS inhibitor L-NAME (100 μmol/L), COX inhibitor INDO (10 μmol/L), sGC inhibitor ODQ (10 μmol/L), or cGMP inhibitor methylene blue (MB) (10 μmol/L) in endothelium-intact artery rings. Arteries were pre-constricted with U46619. N = 6, *p < 0.05, **p < 0.01, ***p < 0.001 vs. control.

**FIGURE 5:**
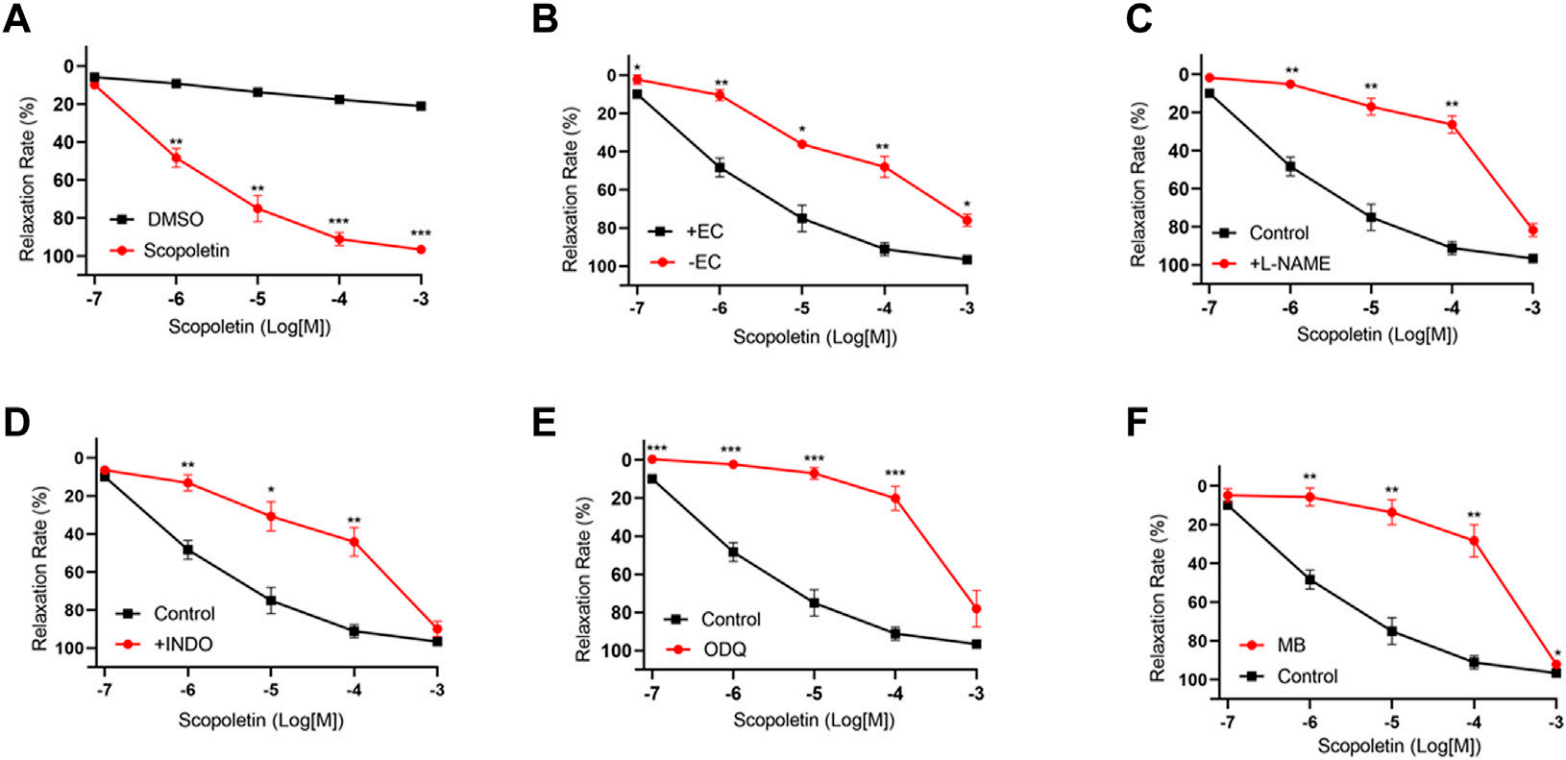
Endothelium-dependent mechanism of the vasorelaxation action of scopoletin. **(A)** Relaxation curve of scopoletin. **(B)** Relaxation curve of scopoletin on endothelium-intact (+EC) and endothelium-denuded (-EC) coronary artery rings. **(C–F)** Relaxation curve of scopoletin on coronary artery ring pretreatment with L-NAME (100 μmol/L), INDO (10 μmol/L), ODQ (10 μmol/L), or MB (10 μmol/L) in endothelium-intact artery rings. Arteries were pre-constricted with U46619. N = 6, *p < 0.05, **p < 0.01, ***p < 0.001 vs. control.

Senkyunolide A, a chemical constituent of *Ligusticum chuanxiong* Hort., was found to relax isolated rat aortas (Chan et al., 2007). In line with the previous finding, this study also shows that senkyunolide A (1 mmol/L) significantly relaxes constricted endothelium-intact coronary artery rings with a R_max_ of 101.77%. However, in the endothelium-denuded coronary artery rings, senkyunolide A R_max_ value decreased to 64.43% (Figures 4A,B). Senkyunolide A relaxation curve and R_max_ value were significantly reduced in coronary artery rings pretreated with L-NAME but not INDO (Figures 4C,D). Furthermore, in the presence of sGC inhibitor ODQ or cGMP inhibitor MB, the relaxation curve and Rmax of senkyunolide A were significantly reduced compared to the control (Figures 4E,F), indicating that senkyunolide A promotes endothelial-dependent vasorelaxation mainly via the eNOS-NO-sGC-cGMP pathway. Coronary arteries are mainly through endothelium-dependent and non-endothelium-dependent dilation. This study revealed that eNOS, sGC, and cGMP inhibitors have limited effect. Senkyunolide A may also play a role in vasodilating through other pathways. Scopoletin, like senkyunolide A, had a potent endothelium-dependent vasorelaxant effect. The R_max_ of scopoletin in endothelium-intact coronary arteries dropped from 96.58% to 75.98% in endothelium-denuded coronary arteries (Figures 5A,B). Scopoletin relaxation curves decreased significantly in the presence of L-NAME and INDO compared to the control (Figures 5C,D). Furthermore, in the presence of ODQ and MB, the relaxation curves for scopoletin were significantly reduced (Figures 5E,F). These findings indicate that scopoletin induces endothelial-dependent relaxation via both the NO and PGI2 pathways.

### 3.7 Vasorelaxation of senkyunolide a and scopoletin via akt-eNOS-NO pathway

**FIGURE 6:**
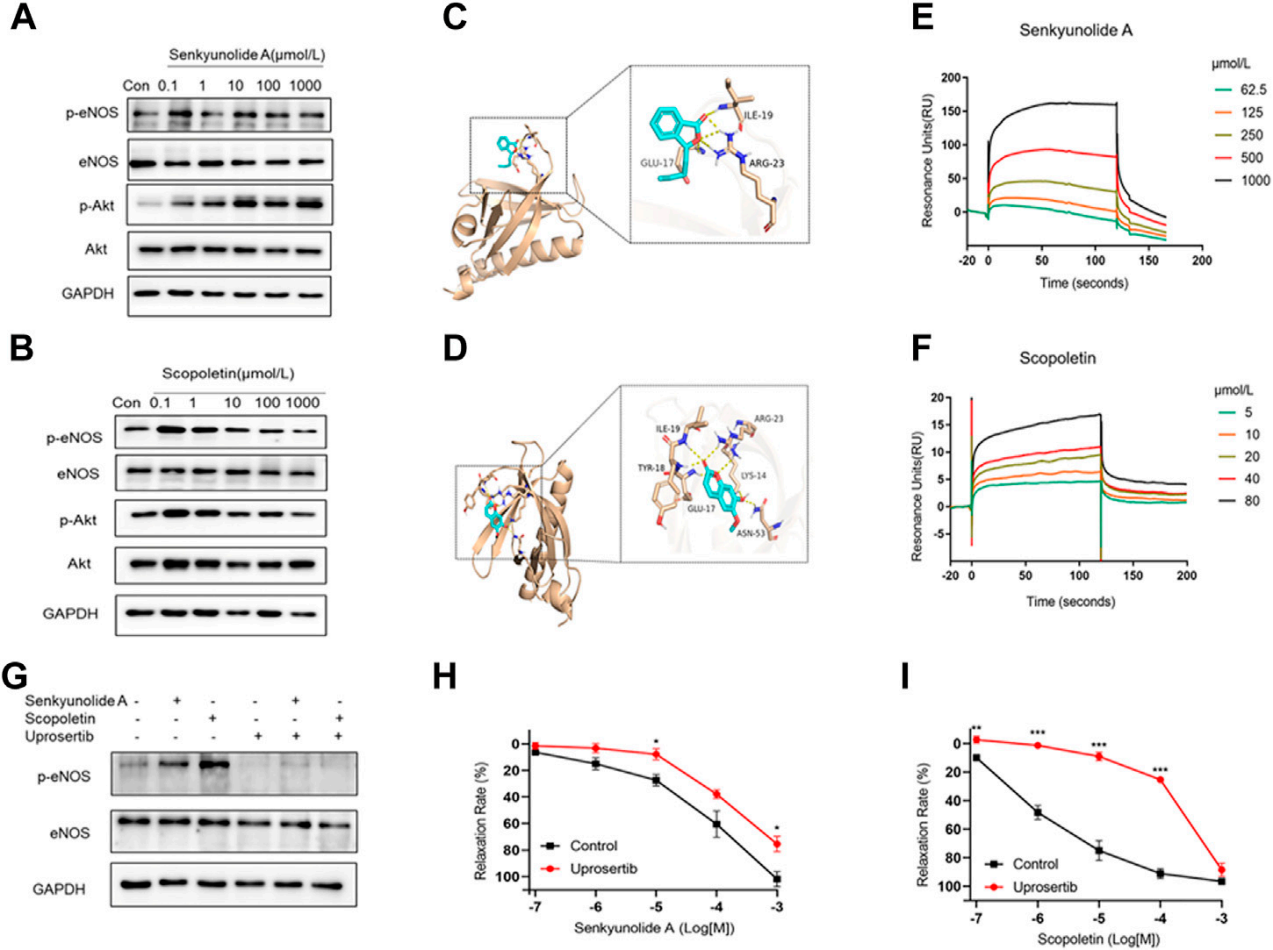
Senkyunolide A and scopoletin induced vasodilation via the Akt-eNOS pathway. **(A–B)** Western blot analysis of p-eNOS, eNOS, p-Akt, and Akt in HUVECs treatment with the indicated concentration of senkyunolide A **(A)** or scopoletin **(B)**. **(C–D)** Prediction of senkyunolide A **(C)** and scopoletin **(D)** binding with Akt by *in silico* molecular docking. **(E–F)** The real-time interactions between senkyunolide A **(E)** or scopoletin **(F)** and Akt were determined by SPR kinetic assay. **(G)** HUVECs were treated with the Akt inhibitor uprosertib (10 μmol/L) for 30 min before exposure to senkyunolide A or scopoletin (100 μmol/L). The regulation effect of senkyunolide A or scopoletin on p-eNOS was measured by Western blot analysis. **(H–I)** The cumulative concentration-response curve of senkyunolide A **(H)** or scopoletin **(I)** in endothelium-intact rings in the presence of uprosertib. N = 3, *p < 0.05, **p < 0.01, ***p < 0.001 vs. control.

The Akt kinase is needed to control the phosphorylation of eNOS and, in turn, NO production (Huang et al., 2020). Thus, the Akt-eNOS-NO endothelium-dependent pathway was studied to determine how senkyunolide A and scopoletin induce endothelium-dependent vasorelaxation. In HUVECs, senkyunolide A or scopoletin treatment increased Akt phosphorylation at Thr308 and eNOS phosphorylation at Ser1177 (Figure 6A–B, Supplementary Figure S1). Following that, *in silico* molecular docking was used to ascertain whether senkyunolide A or scopoletin could bind to critical regulatory sites on Akt, thereby promoting its phosphorylation.

For PDK1 or mTOR2 to phosphorylate Akt, the pleckstrin homology (PH) of Akt must first bind to the lipid second messengers PI (3,4,5) P3 and PI (3,4) P2 (Truebestein et al., 2021). Senkyunolide A and scopoletin both exhibited a strong binding affinity for the Akt1 PH domain (PDB ID: 1H10), with the former having a binding energy of -4.8 kcal/mol and the latter having a binding energy of -5.3 kcal/mol. Additionally, protein-ligand interaction analyses showed that senkyunolide A forms three hydrogen bonds with GLU17, ILE19, and ARG23, while scopoletin forms six hydrogen bonds with LYS14, GLU17, TYR18, ILE19, ARG23, and ASN53 (Figures 6C,D). This indicates that senkyunolide A and scopoletin may exert a regulatory activity on Akt phosphorylation through their interaction with key binding site residues in the PH domain. The SPR biophysical assay was carried out with recombinant Akt protein to investigate the direct interaction between senkyunolide A/scopoletin and Akt. The SPR assay consistently demonstrated that senkyunolide A or scopoletin interacts with Akt in a dose-dependent manner (Figures 6E,F). The value of the equilibrium dissociation constant (*KD*) for scopoletin was determined to be 11.09 μmol/L, while the value for senkyunolide A was 752 μmol/L.

Uprosertib, an inhibitor of Akt, decreased the expression of p-eNOS in HUVECs that had been treated with senkyunolide A and scopoletin (Figure 6G, Supplementary Figure S2). Consistently, in *ex-vivo*, coronary artery rings pretreated with 10 μmol/L of uprosertib, the relaxation curves of senkyunolide A and scopoletin were significantly decreased compared to the negative control (Figures 6H,I), indicating that senkyunolide A and scopoletin trigger endothelium-dependent vasodilation via the activation of the Akt-eNOS-NO signaling pathway.

### 3.8 Vasorelaxation action mechanisms of borneol

**FIGURE 7:**
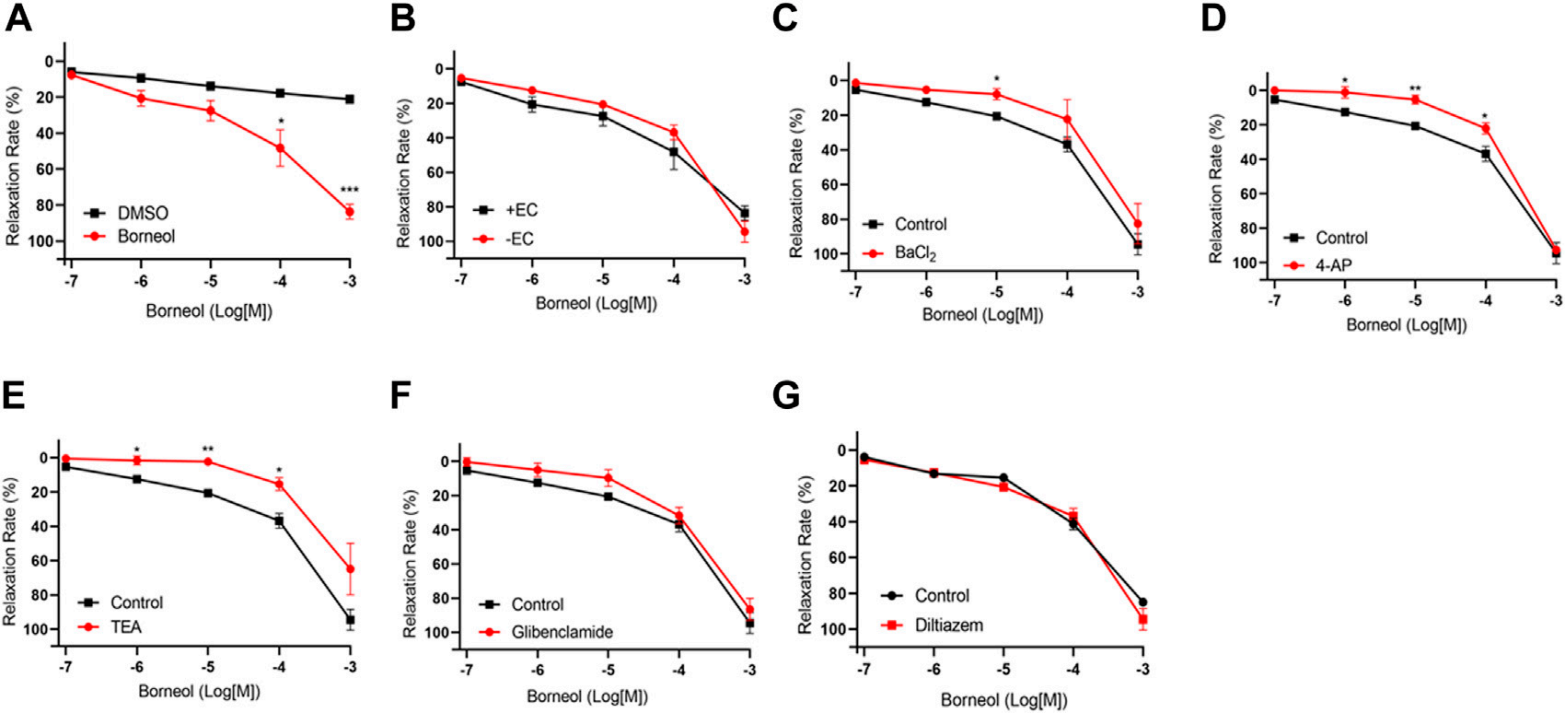
Endothelium-independent mechanism of the vasorelaxation action of borneol. **(A)** Relaxation curve of borneol. **(B)** Relaxation curve of borneol on coronary artery rings with or without endothelium. **(C–G)** Relaxation curve of borneol on endothelium-denuded coronary artery pretreatment with BaCl2 **(C)**, 4-AP **(D)**, TEA **(E)**, glibenclamide **(F)**, or diltiazem **(G)**. N = 6, *p < 0.05, **p < 0.01 vs. control.

The EC_50_ value for borneol-induced concentration-dependent coronary artery ring relaxation was 119.7 μmol/L, and the R_max_ value was 83.63% (Table 3). However, the R_max_ value did not decrease in endothelium-deficient coronary arteries, which shows that the vasorelaxation action of borneol was caused by pathways that do not depend on endothelium (Figures 7A,B). The vasorelaxation effect of borneol was blocked in the presence of potassium channel inhibitors like BaCl_2_, TEA, or 4-AP (Figures 7C–E). However, the vasodilation activity of borneol was unaffected by the K_ATP_ inhibitor glibenclamide or the L-type calcium channel inhibitor diltiazem (Figures 7F,G), indicating that borneol likely stimulates the opening of K_ir_, K_Ca2+_, and K_v_ channels in vascular smooth muscle cells to induce vasorelaxation.

## 4 Discussion

This study reveals the anti-angina pectoris and vasorelaxant effects of Suxiao Jiuxian Pill and clarifies the molecular mechanism of its bioactive constituents. This study reveals that Suxiao Jiuxin Pill alleviates acute myocardial infarction. Senkynolide A, scopoletin, and borneol are Suxiao Jiuxin Pill’s bioactive compounds responsible for coronary artery relaxing actions. Senkynolide A and scopoletin relax coronary arteries by activating the endothelium-dependent Akt-eNOS-NO pathway. Borneol also relaxes coronary artery rings by activating K_ir_, K_Ca2+_, and K_v_ channels in endothelium vascular smooth muscle cells. These findings demonstrate that Suxiao Jiuxin Pill’s anti-AMI effects are primarily mediated via coronary artery relaxation. Furthermore, the active compounds responsible for vasodilation are senkynolide A, scopoletin, and borneol.

Suxiao Jiuxin Pill is a well-known medicine used traditionally in China for the treatment of coronary heart diseases such as angina and acute coronary syndrome (Ren, L. et al., 2018). Sublingual administration of the Suxiao Jiuxin Pill provides rapid relief from angina (Duan et al., 2008). Suxiao Jiuxin Pill improves cardiac function after 5 min of intraperitoneal injection to mimic sublingual administration, which is faster than oral administration. Similarly, Lu *et al.* found that 30 min of duodenum infusion with the Suxiao Jiuxin Pill could attenuate myocardial ischemia, elevate the ST segment, and enhance serum SOD activity in MI dogs (Lu, Z. et al., 2015). These findings indicate that the Suxiao Jiuxin Pill possesses anti-anginal properties.

Coronary heart disease is caused by narrowed and blocked coronary arteries, which deprive the cardiac muscle of oxygen and nutrients (Du et al., 2021). Using LC-MS and GC-MS, 39 non-volatile and volatile compounds were identified in the sera of Suxiao Jiuxin Pill-treated rats. Network pharmacology analysis identified NOS3 and PTGS2 as core acute myocardial ischemia targets. NOS3 and PTGS2 proteins regulate vasodilation by inducing endothelial NO and PGI2 production. For this reason, it was hypothesized that Suxiao Jiuxin Pill’s action on alleviating AMI might partially be due to its ability to induce endothelium-dependent vasorelaxation. Suxiao Jiuxin Pill did induce concentration-dependent vasorelaxant effects on coronary artery rings, with an EC_50_ value of 136.1 ug/mL. He *et al.* found that the Suxiao Jiuxin Pill relaxes the U46619-contracted human internal mammary artery with an EC_50_ of 645.65 ug/ml (Bai et al., 2014). L-NAME treatment prevented Suxiao Jiuxin Pill-induced vasorelaxation in both the coronary and internal mammary arteries. These findings corroborate the NOS3 pathway predicted by network pharmacology and show that the Suxiao Jiuxin Pill induced coronary arteries in a manner dependent on the endothelium.

In this study, 22 compounds from both the botanical material of Suxiao Jiuxin Pill as well as the sera of Suxiao Jiuxin Pill-treated mice were used to identify bioactive compounds responsible for the vasorelaxation effects of Suxiao Jiuxin Pill (Lei et al., 2018). The potential of these compounds to elicit vasorelaxation responses in coronary arteries with intact or denuded endothelium varied. A strong vasodilation response was induced by scopoletin, borneol, and four phthalides: sedanolide, butylphthalide, senkyunolide A, and ligustilide. Recent studies have shown that ligustilide (Chan et al., 2007), borneol (Silva-Filho et al., 2012), and the combination of senkyunolide A and liqustilide (Lei et al., 2018) have vasodilation activity on the aorta and mesenteric artery. This lends credence to the hypothesis that the vasorelaxant effect of Suxiao Jiuxin Pill may be attributed to the presence of these chemical compounds.

Senkyunolide A is a *ligusticum wallichii*-derived phthalide (Lei et al., 2018; Liu, J. L. et al., 2014). Some studies have emphasized the role of senkyunolide A in vasorelaxation. This study reveals that senkyunolide A causes vasorelaxation via an endothelium-dependent pathway. The two essential pathways that regulate endothelium-dependent vasorelaxation are NO/sGC/cGMP and PGI_2_/AC/cAMP (Cohen, 1995). Results from this study show that L-NAME, methylene blue, or ODQ, which are all well-known inhibitors of the NO/sGC/cGMP pathway, inhibited senkyunolide A-induced vasorelaxation. INDO did not affect the relaxations induced by senkynolide A, suggesting that the endothelium-dependent relaxation induced by senkynolide A involves the NO-mediated vasodilatory pathway. Regulation of smooth muscle membrane potential through alteration in K channel activity is a major mechanism of vasodilation and vasoconstriction under both physiological and pathophysiological conditions (Li, X. et al., 2011). The diastolic mechanism of senkyunolide A for ion channels is unknown and needs to be further studied.

Many kinases stimulate the phosphorylation of eNOS at Ser1177 and NO production (Lee et al., 2018). These kinases include Akt, PKA, AMPK, PKG, and CaMKII. In this study, senkyunolide A led to an elevated phosphorylation of Akt-T308 and subsequently eNOS. Moreover, uprosertib, an Akt inhibitor, inhibited both Akt phosphorylation and endothelium-dependent relaxation, demonstrating that senkyunolide A induced vascular tone changes in rat-isolated coronary arteries via the endothelium Akt-eNOS-NO pathway. Senkyunolide A and ligustilide from Suxiao Jiuxin Pill showed Ca^2+^-inhibitory activity by activating CaMKII in VSMCs and causing thoracic aortic relaxation (Lu, Y. et al., 2022). These results suggest that senkyunolide A causes vasorelaxation by increasing CaMKII activity in VSMCs and activating the Akt/eNOS/NO pathway in endothelial cells. Scopoletin is a coumarin derivative isolated from *Liqusticum* chuanxiong Hort. (Ren, D. C. et al., 2007), *Liqusticum* jeholense Nakai et Kitagawa (Zhang, J. L. et al., 1996), and other plants. Scopoletin possesses various pharmacological properties including vasorelaxation, anti-angiogenesis, and anti-inflammation (Pan et al., 2011). In this study, scopoletin-mediated relaxation was inhibited by both L-NAME and INDO, indicating that scopoletin endothelium-dependent relaxation occurs via the NO and PGI_2_ pathways. Scopoletin, like senkyunolide A, stimulates vasorelaxation by activating the Akt-eNOS-NO pathway.

Molecular docking is a molecular bioinformatics technique for predicting the binding mode and the intermolecular interactions between ligands and their target receptor or protein. The pleckstrin homology (PH) domain of Akt is critical in the phosphorylation process of Akt (Truebestein et al., 2021). Both senkyunolide A and scopoletin exhibited high binding affinities for the Akt1 PH domain. Senkyunolide A forms hydrogen bonds with amino acid residues Glu17, ILE19, and ARG23, whiles scopoletin forms hydrogen bonds with amino acid residues LYS14, GLU17, TYR18, ILE19, ARG23, and ASN53. Biophysical characterization study further confirmed the molecular interaction between senkyunolide A or scopoletin and Akt.

The conclusion that scopoletin does not induce endothelium-independent vasorelaxation is inconsistent with the findings of some earlier studies. For instance, a previous study found that scopoletin induces vasorelaxation of the rat thoracic aorta via the blockage of intracellular Ca^2+^ mobilization from noradrenaline-sensitive stores (Oliveira et al., 2001) or inhibits Ca^2+^ release from the sarcoplasmic reticulum (Iizuka et al., 2007). Moreover, EC_50_ values for the vasorelaxation activities of scopoletin in arteries appear distinct. In the rat coronary artery, the EC_50_ value was 1.51 μmol/L, while in the rat mesenteric artery, the EC_50_ value was 112 μmol/L. Also, in rat aortic rings, the EC_50_ value was 180–335 μmol/L. This suggests that scopoletin has tissue-specific sensitivity effects, and it is extremely treatment-sensitive to the coronary artery. Thus, scopoletin might be a potent drug candidate for the treatment of myocardial ischemia.

Borneol is another compound found in Suxiao Jiuxin Pill. Findings from this study indicate that borneol induces vasorelaxation activity via endothelium-independent pathways. Endothelium-independent vasodilation is achieved mainly by antagonizing or activating various ion channels on the VSMCs membrane, such as the K^+^ channel and Ca^2+^ channel. In VSMCs, four distinct types of K^+^ channels have been identified: K_Ca2+_, K_V_, K_ATP_, and K_ir_ (Dogan et al., 2019). Modulating K^+^ channel opening results in VSMCs membrane hyperpolarization and voltage-gated calcium channel closure, which inhibits Ca^2+^ influx and subsequent vasodilation (Yao et al., 2016). Indeed, pretreatment with BaCl_2_, TEA, or 4-AP, but not Glibenclamide, reduced borneol’s vasorelaxation effect, demonstrating that borneol’s vasodilation is primarily triggered by the opening of K_v_, K_Ca2+,_ and K_ir_ channels. This result is consistent with a previous study that found that (-)-borneol induces an endothelium-independent vasorelaxant effect by a reduction in Ca^2+^ influx and activation of K_v_, K_Ca2+,_ and K_ir_ (Silva-Filho et al., 2012). In contrast, (-)-borneol relaxed rat aortic rings via NO and PGI_2_-mediated endothelium-dependent vasorelaxation and directly impacted VSMCs via K_ATP_ channels (Santos et al., 2019). Thus, the mechanism of action of (-)-borneol-induced vasodilation is complicated and requires further investigation.

Overall, Suxiao Jiuxin Pill improves cardiac function in acute myocardial ischemia and causes vasorelaxation of the coronary artery. In addition, it was revealed that scopoletin, phthalides, and borneol are the primary bioactive compounds of Suxiao Jiuxin Pill that contribute to its vasodilatory effects. Vasorelaxation induced by senkyunolide A and scopoletin is through the endothelium-dependent Akt-eNOS-NO pathway. Borneol triggers vasodilation in VSMCs by opening potassium channels.

## Data Availability

The datasets presented in this study can be found in online repositories. The names of the repository/repositories and accession number(s) can be found in the article/Supplementary Material.

## Abbreviations

4-AP, 4-Aminopyridine; Ach, Acetylcholine chloride; Akt, Protein kinase B; AMI, Acute myocardial infarction; DMSO, Dimethyl sulfoxide; ECG, Electrocardiogram; EF, Ejection fraction; eNOS, Endothelial nitric oxide synthase; ESI-MS, Electrospray ionization mass spectrometry; FS, Fractional shortening; GC-MS, Gas chromatography-mass spectrometry; Gli, Glibenclamide; HUVEC, Human umbilical vein endothelial cells; INDO, Indomethacin; KCl, Potassium chloride; L-NAME, N^G^-nitro-l-arginine methyl ester; LC-MS, Liquid chromatography-mass spectrometry; LVID, left ventricular internal dimension diastole; MB, Methylene blue; NO, Nitric oxide; ODQ, 1H- [1,2,4] oxadiazolo [4,2-α] quinoxalin-1-one; PGI_2_, Prostacyclin; SJP, Suxiao Jiuxin Pill; SPR:Surfac, e plasmon resonance; TEA, Tetraethylammonium; U46619, 9, 11-Didexy-11α, 9α-epoxymethanoprostaglandin F2α.
