# Supplementary material for: Suxiao Jiuxin Pill attenuates acute myocardial ischemia via regulation of coronary artery tone
Source: Front Pharmacol. 2023 May 10;14:1104243. doi: 10.3389/fphar.2023.1104243 (PMC10206061; doi:10.3389/fphar.2023.1104243)
Supplement: Supplementary file 1 [file Presentation1.PPTX]

## Slide 1
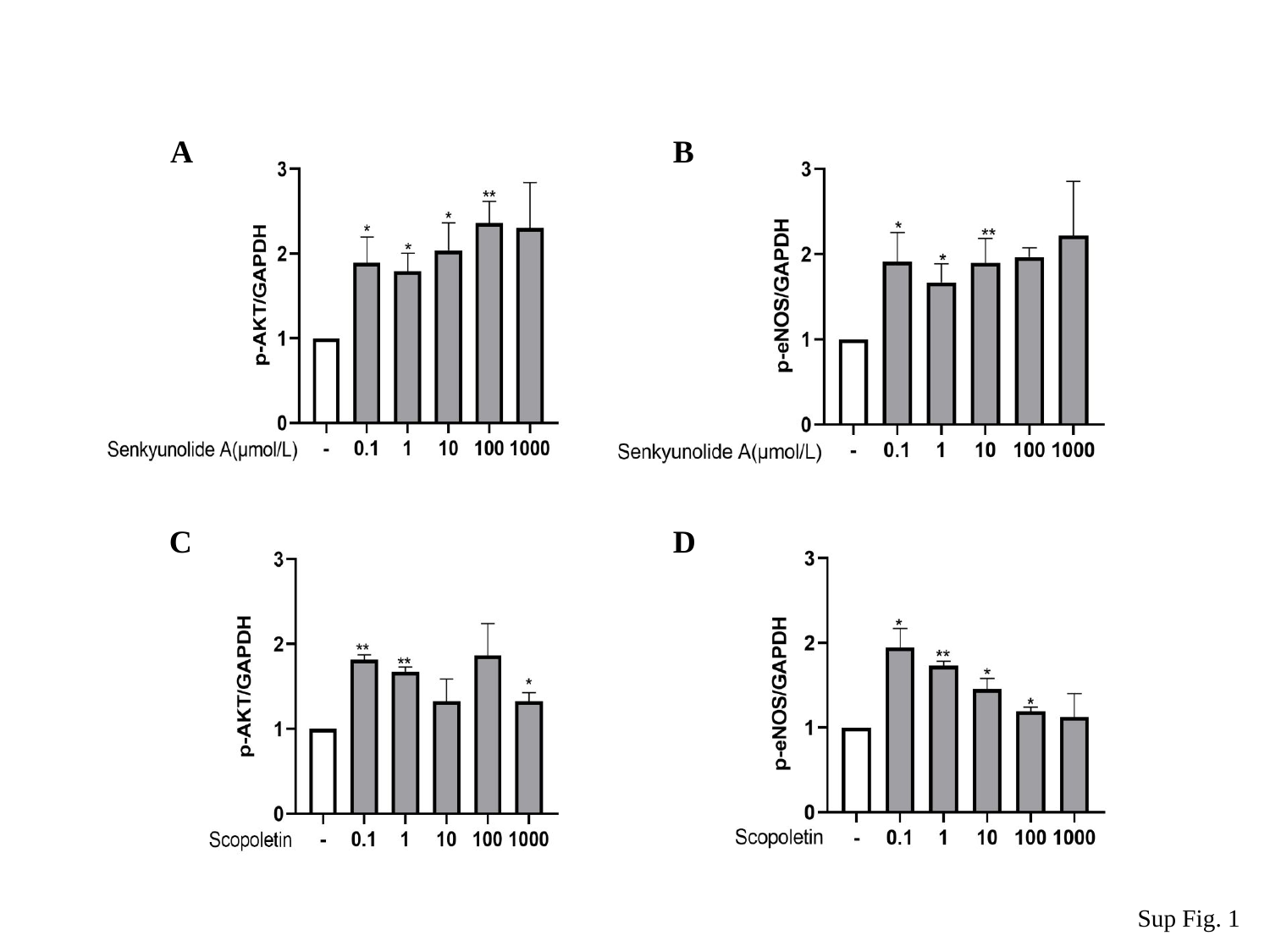

A
B
C
D
Sup Fig. 1

## Slide 2
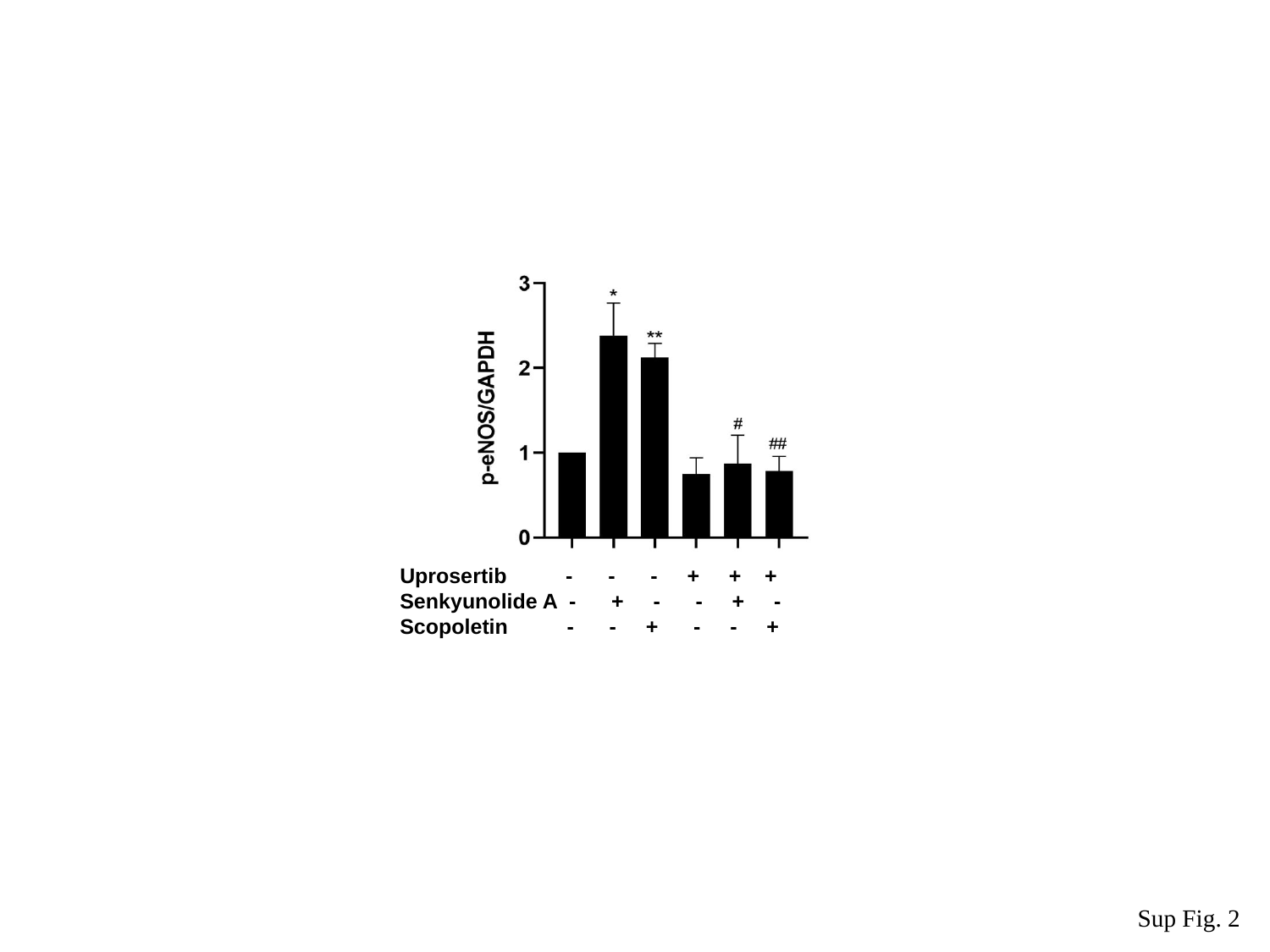

Uprosertib - - - + + +
Senkyunolide A - + - - + -
Scopoletin - - + - - +
Sup Fig. 2

## Slide 3
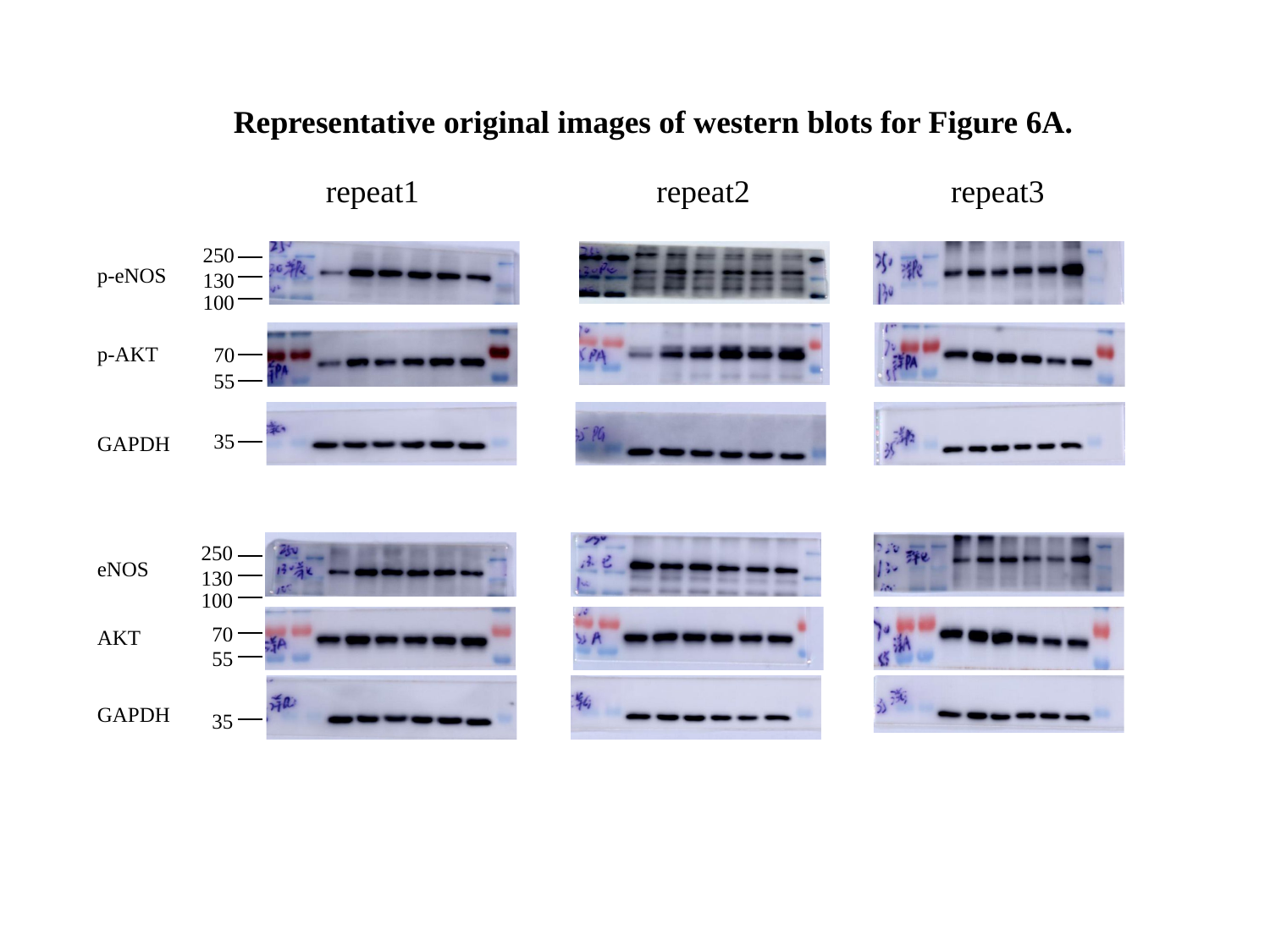

Representative original images of western blots for Figure 6A.
repeat1
repeat2
repeat3
250
p-eNOS
130
100
p-AKT
70
55
35
GAPDH
250
eNOS
130
100
70
AKT
55
GAPDH
35

## Slide 4
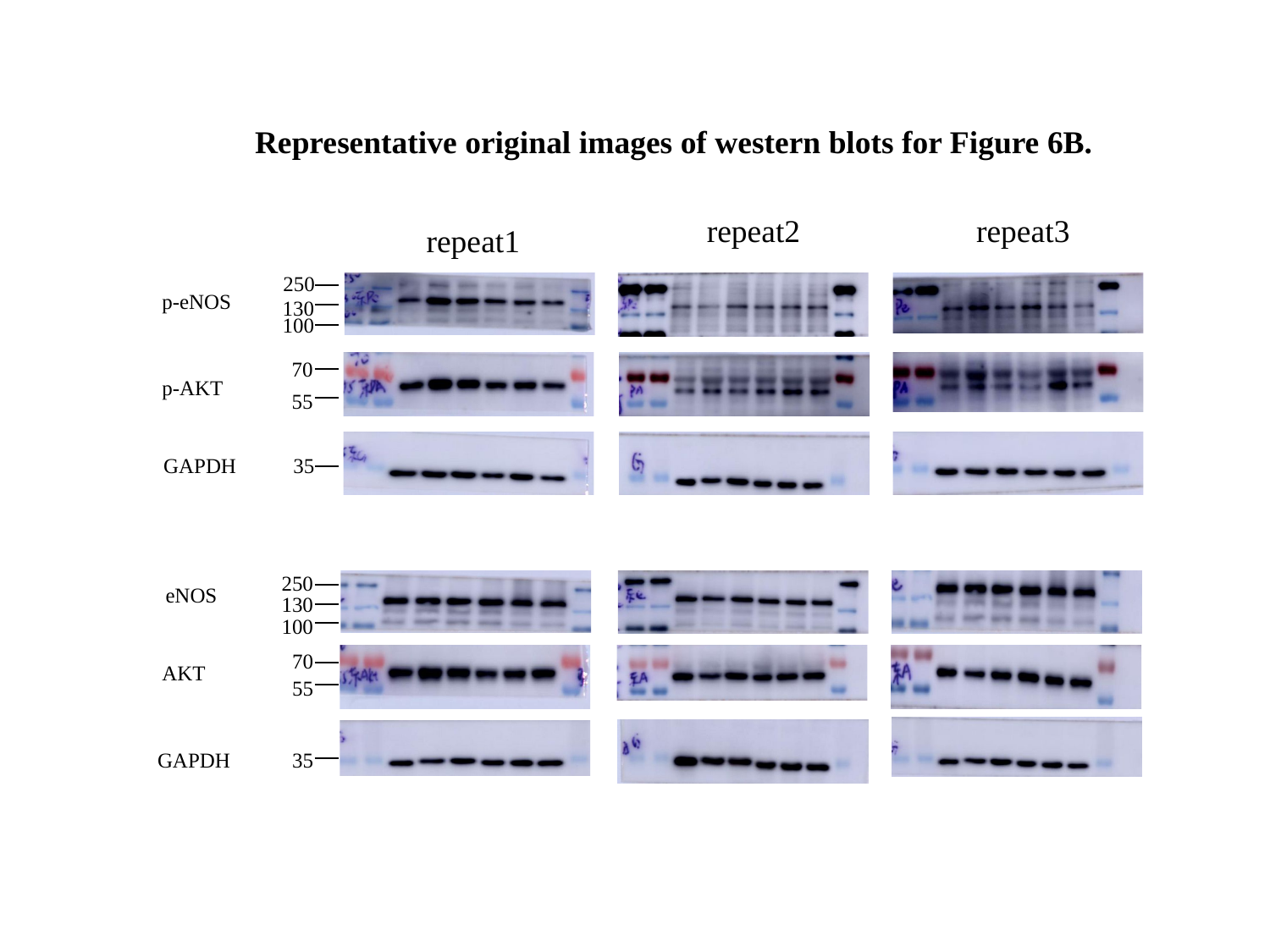

Representative original images of western blots for Figure 6B.
repeat2
repeat3
repeat1
250
p-eNOS
130
100
 70
p-AKT
 55
GAPDH
35
250
eNOS
130
100
70
AKT
55
35
GAPDH

## Slide 5
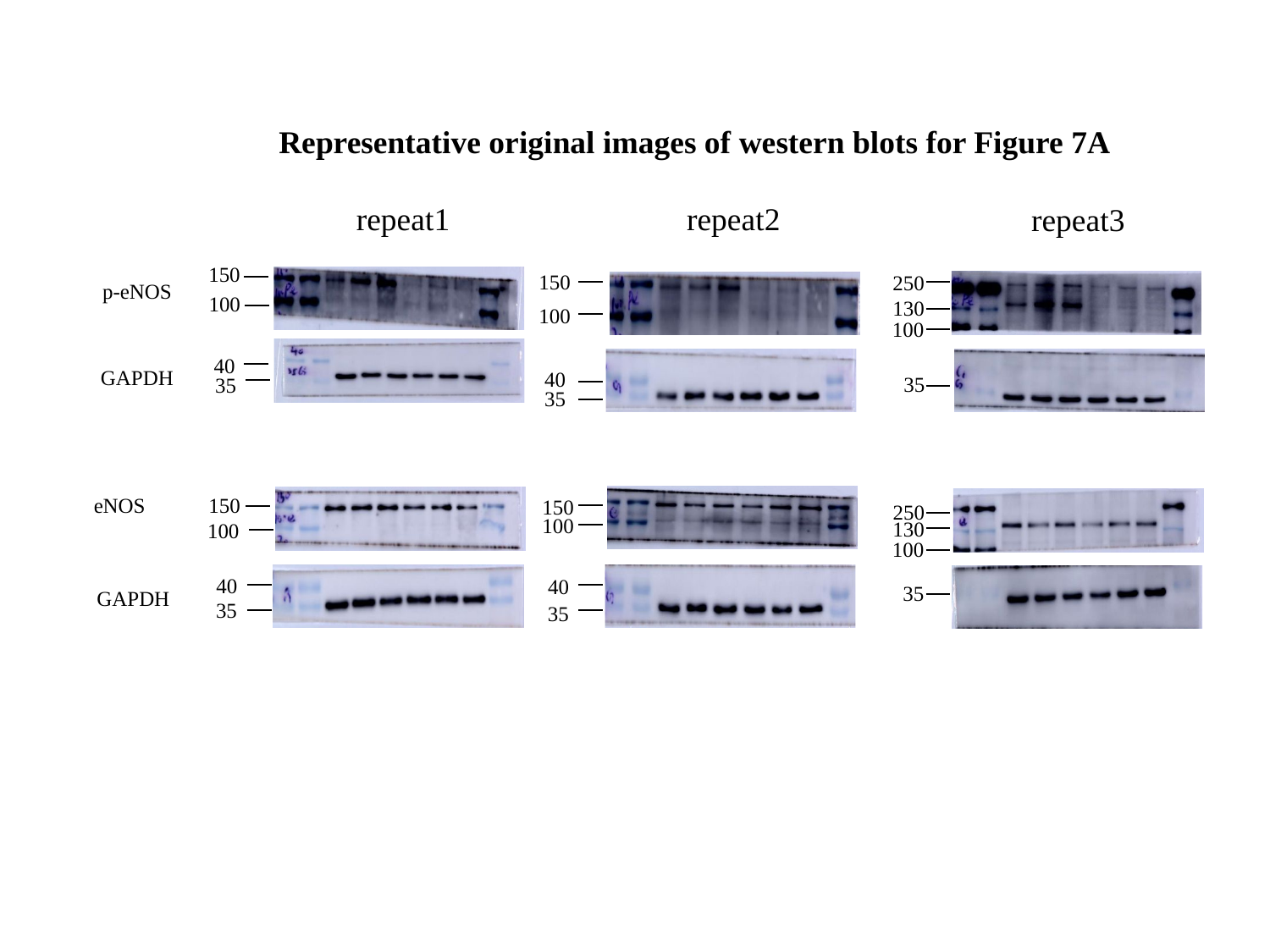

Representative original images of western blots for Figure 7A
repeat1
repeat2
repeat3
150
150
250
p-eNOS
100
130
100
100
40
GAPDH
40
35
35
35
150
eNOS
150
250
100
130
100
100
40
40
35
GAPDH
35
35
